# A Pilot Study Evaluating the Prevalence of Cervical Spine Dysfunction Among Students of Dentistry at the Medical University

**DOI:** 10.3389/fneur.2020.00200

**Published:** 2020-03-31

**Authors:** Joanna Kuć, Małgorzata Żendzian-Piotrowska

**Affiliations:** ^1^Department of Prosthodontics, Medical University of Bialystok, Białystok, Poland; ^2^Department of Hygiene, Epidemiology and Ergonomics, Medical University of Bialystok, Białystok, Poland

**Keywords:** cervical spine dysfunction, chronic pain, graded chronic pain scale, neck disability index, neck pain, orofacial pain, perceived stress scale

## Abstract

**Introduction:** Cervical pain affects most people at some stage, especially middle-aged. It is one of the symptoms of cervical spine dysfunction. The prevalence of neck pain varies and depends on the population studied.

**Aim:** The aim of the study was to assess the prevalence of cervical spine dysfunction among dentistry students from a medical university.

**Materials and Methods:** The study was conducted on a group of 112 randomly chosen, generally healthy students (73 women and 39 men) of the faculty of medicine, division of dentistry at a medical university, aged 20–32, average 22.88 ± 2.7. The survey was based on a questionnaire about possible symptoms and disorders of the cervical spine in the 6 preceeding months. A bodychart was used to visualize ailments in relation to the scheme of human body, and a Graded Chronic Pain Scale was applied to assess levels of pain. Additionally, the Perceived Stress Scale and Neck Disability Index were evaluated.

**Results:** With respect to the questionnaire about possible symptoms of cervical spine disorders in the 6 preceeding months, 22.32% of students declared headaches 2–3 times a week, and 45.53% 2–3 times a month. 42.85% of the participants reported difficulties with concentration, 56.25% showed attention issues, and 25% had problems with memory. Moreover, 9.82% of the subjects suffered from depression, and 27.67% declared mood disorders. The bodychart revealed the occurrence of pain within the cervical spine in 47.32% of the respondents. 31.25% of students declared discomfort in the suboccipital area. In 57.14% of people, low intensity of chronic pain without functional disorders was noted. A moderate level of stress was reported in 58.03% of students. Mild cervical spine disorders were found in 53.57% of cases.

**Conclusion:** The relatively high prevalence of symptoms of the cervical spine dysfunction, chronic pain, stress, and neck disability among young people may indicate that numerous spinal disorders identified in dentists result not only from their profession, in which spine is significantly overloaded, but also individual predispositions including biopsychosocial profile and the changing lifestyle habits of young people.

## Introduction

Neck pain is one of the most common symptoms of musculoskeletal disorders (1). After back pain it is the second most widespread disorder associated with spine dysfunction experienced by every age group, including children and adolescents (2, 3). Cervical pain is considered the fourth cause of the inefficiency (Global Burden of Disease Study 2010) (1, 4, 5). About 60–80% of professionally active people experience a relapse within 1 year of the onset of the first symptoms (5). The prevalence of neck pain varies and depends on the studied population. It may fluctuate between 6 and 22% or concern 1.5–75% of people (6). It affects 15.1% of the population in the United States, 20.3–24% in Brazil, 19.5% in Spain, 20.4% in Greece, 48.7% in China, and 56.9% in Sri Lanka (4, 7). On average, this disorder affects about 30–50% of the total population (1). The incidence of ailments is significantly higher in women than in men, amounting to 27.2 and 17.4%, respectively (2). It was estimated that as much as 67% of the world population experience neck pain at a certain stage of their lives (2). Due to the chronic nature of the disease, it is important to periodically verify the risk factors, apply preventative measures, and in numerous cases make an early diagnosis (4).

Amongst the main causes of neck pain there are static positions with constantly repeated movements, especially cervical flexion or strong arm movements, as well as prolonged sitting position (8). Ariëns et al. suggested a relationship between neck pain and the degree of cervical flexion (8). The authors indicate an increased risk of pain in people working with a neck flexion of at least 20° for 70% of their total working time (8). On the other hand, functioning in a similar position for a shorter time (25–50% or 50–60% of the working time) does not pose a threat (8). Surprisingly low exposure was achieved by the aforementioned researcher in the bending range ≥45°. In this case, the subjects remained in the said position for short periods of time (<5%, 5–10%, >10% of their total working time) (8). The relationship between cervical spine rotation and pain is not clear (8). A statistically significant positive relationship was observed by Dartqiues et al. (9). However, Musson et al. did not observe such dependency (*p* > 0.05) (10). Ariëns et al. also noted a directly proportional relationship between neck pain and sitting position (8). For people remaining in a sitting position for over 95% of their working time, the risk of cervical spine pain is twice as high as in those who have never worked in this position (8). Ariëns et al. indicated the need to limit the time spent in static positions, thereby promoting the adoption of a more dynamic lifestyle (8). It is commonly believed that there is a relationship between cervical spine pain and spine extension, torsions or torso slips, functioning of the shoulder girdle, lifting heavy objects, and an incorrectly adjusted workplace (11). In order to assess the physical exposure, it is recommended to analyze for the level of daily mechanical load, verify the time of overload exposure, and measure the frequency of repeating of a given performed activity (11).

The aim of this study was to evaluate the prevalence of cervical spine dysfunction among dentistry students from a medical university. The intensity of the experienced stress and cervical spine disability were analyzed.

## Materials and Methods

### The Subjects and Sample Size

The study was conducted on a group of 112 randomly chosen, generally healthy students (73 women and 39 men) of the faculty of medicine, division of dentistry of a medical university, aged 20–32, average 22.88 ± 2.7 (Me_total study group_ = 22, Me_group of women_ = 22.99, Me_group of men_ = 22.67). The assessment included people in their second to fifth year of studies. The research was conducted in the period from January to April 2018.

### General Description of the Method

**Figure 1:**
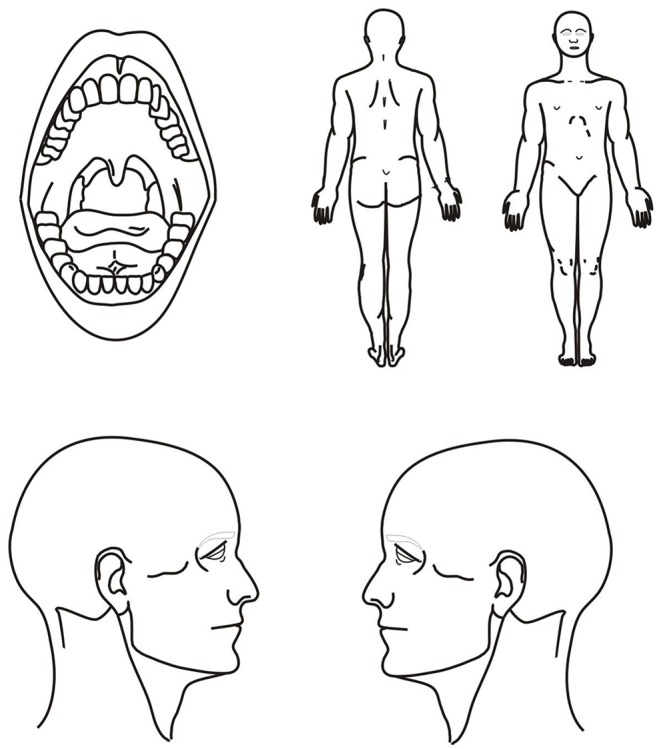
Bodychart adapted from Kuć et al. (12).

The survey was conducted with the use of the following:

- Questionnaire concerning possible symptoms and disorders of the cervical spine in the 6 preceeding months
- Bodychart (pain drawing) enabling the visualization of ailments in relation to the human body scheme (Figure 1)
- Graded Chronic Pain Scale version 2.0 (GCPS v. 2.0)
- Perceived Stress Scale (PSS−10)
- Neck Disability Index (NDI).

Response rate to questionnaires was 100%.

### Statistical Analysis

A statistical analysis was performed using Statistica 13.1 (Statsoft Inc. USA) and PQStat 1.8.0 Software. A Chi-square test of independence for 2 × 2 table was used to compare the frequency of pain locations with respect to gender. For GCPS v. 2.0, PSS-10 and NDI RxC contingency tables were used. For small sized sample (expected number of frequencies ≤ 5), Fisher's exact one-tailed test was applied. Differences with *p* < 0.05 were considered statistically significant. For Fisher's exact unilateral test, *post hoc* power analysis was performed using G Power v.3.1.9.4 Software. Power (1-β) was calculated as the function of the population effect size, α and a number of cases (n).

A multiple-comparison correction was applied. To control the familywise error rate and get the Bonferroni critical value (Bonferroni adjusted *p*-value), *p* = 0.05 was divided by the number of tests (*n* = 33, Tables 1, 2; *n* = 53, Tables 3, 4). To control the false discovery rate Benjamini-Hochberg procedure was performed.

**Table 1:** Symptoms and disorders related to the cervical spine noted in the 6 preceeding months in the entire study group (*n* = 112), the group of women (*n* = 73), and the group of men (*n* = 39).

				**Comparison with respect to gender**					
**Part I** **Symptoms and disorders of the cervical spine**	**Entire** **group**	**Group of women**	**Group of men**	**Pearson Chi-Square test (χ2 test)**			**Fisher's Exact Unilateral Test**		**Benjamini-Hochberg Correction**
	***n* = 112**	***n* = 73**	***n* = 39**	**Chi^**2**^**	**df**	***p*-value**	***p*-value**	**1-β**	***p*-value**
Headache (2–3 times a week)	25 (22.32%)	21 (28.77%)	4 (10.26%)	5.023339	1	0.02510^*^	0.01943^*^	0.6915863	0.21070
Headache (2–3 times a month)	51 (45.54%)	35 (47.95%)	16 (41.03%)	0.490753	1	0.48359	0.30868	0.1281030	0.55126
Stiffness of the cervical spine	31 (27.68%)	24 (32.88%)	7 (17.95%)	2.829839	1	0.09253	0.07001	0.4423988	0.34519
Limitation in the mobility of the cervical spine	13 (11.61%)	11 (15.07%)	2 (5.13%)	2.448076	1	0.11767	0.10125	0.3403579	0.39431
Transient torticollis	4 (3.57%)	3 (4.11%)	1 (2.56%)	0.176300	1	0.67457	0.56580	0.0111250	0.62051
Dizziness	34 (30.36%)	28 (38.36%)	6 (15.38%)	6.344721	1	0.01177^*^	0.00908^*^	0.7852335	0.21070
Balance disorders	14 (12.50%)	12 (16.44%)	2 (5.13%)	2.972954	1	0.08467	0.07225	0.4189811	0.34519
Speech disorders	6 (5.36%)	5 (6.85%)	1 (2.56%)	0.920647	1	0.33731	0.31601	0.0886361	0.55126
Dysphagia	2 (1.79%)	1 (1.37%)	1 (2.56%)	0.206712	1	0.64936	0.57722	0.0353574	0.62051
Double vision	3 (2.68%)	2 (2.74%)	1 (2.56%)	0.003008	1	0.95626	0.72257	0.0014183	0.72257
Sudden falls	0 (0.00%)	0 (0.00%)	0 (0.00%)	-	-	-	-	-	-
Coordination disorders	5 (4.46%)	4 (5.48%)	1 (2.56%)	0.506562	1	0.47663	0.42838	0.0388787	0.21070
Muscle tension decrease	3 (2.68%)	3 (4.11%)	0 (0.00%)	1.646852	1	0.19939	0.27289	0.0303921	0.57693
Muscle tension increase	20 (17.86%)	15 (20.55%)	5 (12.82%)	1.034804	1	0.30903	0.22680	0.1817952	0.55126
Chronic muscle fatigue	11 (9.82%)	6 (8.22%)	5 (12.82%)	0.607658	1	0.43567	0.32050	0.1323107	0.51328
Nausea	18 (16.07%)	14 (19.18%)	4 (10.26%)	1.500022	1	0.22067	0.17048	0.2373981	0.55126
Vomiting	7 (6.25%)	3 (4.11%)	4 (10.26%)	1.639152	1	0.20044	0.18997	0.2738398	0.45382
Numbness of the upper limbs	18 (16.07%)	15 (20.55%)	3 (7.69%)	3.114529	1	0.07760	0.06326	0.4585820	0.45382
Numbness of the lower limbs	10 (8.93%)	6 (8.22%)	4 (10.26%)	0.129744	1	0.71870	0.48301	0.0653263	0.34519
Nystagmus	1 (0.89%)	1 (1.37%)	0 (0.00%)	0.539060	1	0.46282	0.65179	0.0000669	0.57693
Neuralgia of the trigeminal nerve	1 (0.89%)	0 (0.00%)	1 (2.56%)	1.888658	1	0.16935	0.34821	0.0775966	0.66731
Facial nerve palsy	0 (0.00%)	0 (0.00%)	0 (0.00%)	-	-	-	-	-	-
Allergy	28 (25.00%)	17 (23.29%)	11(28.21%)	0.327831	1	0.56694	0.36193	0.0314230	0.55582
Asthma	2 (1.79%)	0 (0.00%)	2 (5.13%)	3.811655	1	0.05090	0.11921	0.3234556	0.39431

* p < 0.05 statistical significance, adj

**p < 0.00116279 statistical significance adjusted to Bonferroni correction, (1-β) –the power of a statistical test.

**Table 2:** Symptoms and disorders related to the cervical spine noted in the 6 preceeding months in the entire study group (*n* = 112), the group of women (*n* = 73), and the group of men (*n* = 39).

				**Comparison with respect to gender**					
**Part II** **Symptoms and disorders of the cervical spine**	**Entire** **group**	**Group of women**	**Group of men**	**Pearson Chi-Square test (*******χ*******2 test)**			**Fisher's exact unilateral test**		**Benjamini-Hochberg correction**
	***n* = 112**	***n* = 73**	***n* = 39**	**Chi^**2**^**	**df**	***p*-value**	***p*-value**	**1-β**	***p*-value**
Transient paresis of upper limbs	0 (0.00%)	0 (0.00%)	0 (0.00%)	-	-	-	-	-	-
Transient paresis of the lower limbs	0 (0.00%)	0 (0.00%)	0 (0.00%)	-	-	-	-	-	-
Concentration disorders	48 (42.86%)	34 (46.58%)	14 (35.90%)	1.183468	1	0.27665	0.18766	0.2288210	0.45382
Difficulties with attention	63 (56.25%)	43 (58.90%)	20 (51.28%)	0.600086	1	0.43855	0.28230	0.1432828	0.55126
Difficulties with memory	28 (25%)	19 (26.03%)	9 (23.08%)	0.118019	1	0.73119	0.45916	0.0631133	0.57693
Tinnitus	8 (7.14%)	6 (8.22%)	2 (5.13%)	0.366161	1	0.54510	0.42720	0.0608361	0.57693
Migraine	19 (16.96%)	13 (17.81%)	6 (15.38%)	0.105997	1	0.74475	0.48286	0.0527239	0.57693
Ailments within the temporomandibular joint	33 (29.46%)	23 (31.51%)	10 (25.64%)	0.420845	1	0.51652	0.33616	0.1110880	0.55456
Pain in the region of the temporal muscles	18 (16.07%)	15 (20.55%)	3 (7.69%)	3.114529	1	0.07760	0.06326	0.4585820	0.34519
Pain in the area of the masseter muscles	22 (19.64%)	17 (23.29%)	5 (12.82%)	1.764403	1	0.18408	0.13970	0.2883388	0.42908
Numbness of individual fingers	16 (14.29%)	13 (17.81%)	3 (7.69%)	2.124341	1	0.14498	0.11815	0.3125892	0.39431
Pain in the back of the head	14 (12.61%)	5 (6.94%)	9 (23.08%)	5.973413	1	0.01452^*^	0.01774^*^	0.6797027	0.21070
Grinding of the teeth	30 (26.79%)	17 (23.29%)	13 (33.33%)	1.308060	1	0.25275	0.17845	0.2336447	0.45382
Burning tongue	4 (3.57%)	2 (2.74%)	2 (5.13%)	0.421080	1	0.51640	0.43420	0.0959709	0.57693
Hypersensitivity to touch within the face	1 (0.89%)	1 (1.37%)	0 (0.00%)	0.539060	1	0.46282	0.65179	0.0000669	0.66731
Myopia	47 (41.96%)	30 (41.10 %)	17 (43.59%)	0.064914	1	0.79889	0.47724	0.0569304	0.57693
Foresight	6 (5.36%)	5 (6.85%)	1 (2.56%)	0.920647	1	0.33731	0.31601	0.0886361	0.55126
Astigmatism	17 (15.18%)	14 (19.18%)	3 (7.69%)	2.604675	1	0.10655	0.08709	0.3844925	0.37449
Mood disorders	31 (27.68%)	24 (32.88%)	7 (17.95%)	2.829839	1	0.09253	0.07001	0.4423988	0.34519
Depression	11 (9.82%)	7 (9.59%)	4 (10.26%)	0.012783	1	0.90998	0.57587	0.0381846	0.62051
Stress	86 (76.79%)	61(83.56%)	25 (64.10%)	5.399807	1	0.02014^*^	0.01960^*^	0.6590684	0.21070
Breathing problems	8 (7.14%)	6 (8.22%)	2 (5.13%)	0.366161	1	0.54510	0.42720	0.0608361	0.57693
Gastric problems	27 (24.11)	18 (24.66%)	9 (23.08%)	0.034712	1	0.85220	0.52305	0.0457795	0.60787

* p < 0.05 statistical significance, adj **p < 0.00116279 statistical significance adjusted to Bonferroni correction, (1-β) –the power of a statistical test.

**Table 3:** Pain location with respect to the bodychart (Pain Drawing) noted in the entire study group (*n* = 112), the group of women (*n* = 73) and the group of men (*n* = 39).

				**Comparison with respect to gender**					
**Part I** **Pain location**	**Entire** **group**	**Group of women**	**Group of men**	**Pearson Chi-Square test (χ2 test)**			**Fisher's exact unilateral test**		**Benjamini-Hochberg correction**
	***n* = 112**	***n* = 73**	***n* = 39**	**Chi^**2**^**	**df**	***p*-value**	***p*-value**	**1-β**	***p*-value**
Cervical spine cx	53 (47.32%)	39 (53.42%)	14 (35.90%)	3.132591	1	0.07674	0.05760	0.4773586	0.53292
Suboccipital area	35 (31.25%)	25 (34.25%)	10 (25.64%)	0.8762015	1	0.34924	0.23655	0.1767332	0.68345
Thoracic spine th	21 (18.75%)	11 (15.07%)	10 (25.64%)	1.865103	1	0.17204	0.13371	0.2940731	0.54513
Lumbar spine lx	52 (46.43%)	38(52.05%)	14(35.90%)	2.668036	1	0.10238	0.07527	0.4231058	0.53292
Temporal muscle right	34 (30.36%)	27 (36.99%)	7 (17.95%)	4.357683	1	0.03684^*^	0.02851^*^	0.6157159	0.53292
Temporal muscle left	34 (30.36%)	28 (38.36%)	6 (15.38%)	6.344721	1	0.01177^*^	0.00908^*^	0.7852335	0.48124
Masseter muscle right	16 (14.29%)	13 (17.81%)	3 (7.69%)	2.124341	1	0.14498	0.11815	0.3125892	0.53292
Masseter muscle left	16 (14.29%)	14 (19.18%)	2 (5.13%)	4.097881	1	0.04294^*^	0.03530^*^	0.5784283	0.53292
Sternocleidomastoid muscle right	9 (8.04%)	8 (10.96%)	1 (2.56%)	2.424078	1	0.11948	0.11308	0.3243315	0.53292
Sternocleidomastoid muscle left	8 (7.21%)	6 (8.22%)	2 (5.13%)	0.326525	1	0.56771	0.44172	0.0579494	0.68345
Temporomandibular joint left	8 (7.14%)	6 (8.22%)	2 (5.13%)	0.366161	1	0.54510	0.42720	0.0579494	0.68345
Temporomandibular joint right	8 (7.14%)	7 (9.59%)	1 (2.56%)	1.891330	1	0.16905	0.16191	0.2373952	0.61295
Right shoulder	26 (23.21%)	21 (28.77%)	5 (12.82%)	3.626356	1	0.05687	0.04439	0.5417047	0.53292
Left shoulder	19 (16.96%)	14 (19.18%)	5 (12.82%)	0.729375	1	0.39309	0.28227	0.1378466	0.68345
Chest	1 (0.89%)	1 (1.37%)	0 (0%)	0.539059	1	0.46282	0.65179	0.0000669	0.68345
Right groin, right hip joint	3 (2.68%)	1 (1.37%)	2 (5.13%)	1.377369	1	0.24055	0.27743	0.1791129	0.68345
Left groin, left hip joint	0 (0%)	0 (0%)	0 (0%)	-	-	-	-	-	-
Right knee	5 (4.46%)	5 (6.85%)	0 (0%)	2.796057	1	0.09450	0.11196	0.2325498	0.53292
Left knee	2 (1.79%)	2 (2.74%)	0 (0%)	1.087920	1	0.29693	0.42278	0.0038946	0.68345
Teeth I quadrant	4 (3.57%)	3 (4.11%)	1 (2.56%)	0.157159	1	0.69179	0.57637	0.0111250	0.68345
Teeth II quadrant	2 (1.79%)	1 (1.37%)	1 (2.56%)	0.206712	1	0.64936	0.57722	0.0353574	0.68345
Teeth III quadrant	1 (0.89%)	1 (1.37%)	0 (0%)	0.539059	1	0.46282	0.65179	0.0000669	0.68345
Teeth IV quadrant	5 (4.46%)	4 (5.48%)	1 (2.56%)	0.506562	1	0.47663	0.42838	0.0388787	0.68345
Right eye	2 (1.79%)	0 (0%)	2 (5.13%)	3.811655	1	0.05090	0.11921	0.3234556	0.53292
Left eye	4 (3.57%)	1 (1.37%)	3 (7.69%)	2.950474	1	0.08585	0.12066	0.3824161	0.53292
Right supraorbital area	7 (6.25%)	5 (6.85%)	2 (5.13%)	0.128509	1	0.71998	0.53545	0.0328257	0.68345
Left supraorbital area	7 (6.25%)	5 (6.85%)	2 (5.13%)	0.128509	1	0.71998	0.53545	0.0328257	0.68345
Left retromolar area	4 (3.57%)	3 (4.11%)	1 (2.56%)	0.176299	1	0.67457	0.56580	0.0111250	0.68345
Right retromolar area	5 (4.46%)	4 (5.48%)	1 (2.56%)	0.506562	1	0.47663	0.42838	0.0388787	0.68345

* p < 0.05 statistical significance, adj **p < 0.0009434 statistical significance adjusted to Bonferroni correction, (1-β) –the power of a statistical test.

**Table 4:** Pain location with respect to the bodychart (Pain Drawing) noted in the entire study group (*n* = 112), the group of women (*n* = 73) and the group of men (*n* = 39).

				**Comparison with respect to gender**					
**Part II** **Pain location**	**Entire** **group**	**Group of women**	**Group of men**	**Pearson Chi-Square test (χ2 test)**			**Fisher's Exact Unilateral Test**		**Benjamini-Hochberg Correction**
	***N* = 112**	***N* = 73**	***n* = 39**	**Chi^**2**^**	**df**	***p*-value**	***p*-value**	**1-β**	***p*-value**
Right side of the lips	3 (2.68%)	2 (2.74%)	1 (2.56%)	0.003007	1	0.95626	0.72257	0.0014183	0.72257
Left side of the lips	2 (1.79%)	1 (1.37%)	1 (2.56%)	0.206712	1	0.64936	0.57722	0.0353574	0.68345
Trachea region	2 (1.79%)	0 (0%)	2 (5.13%)	3.811655	1	0.05090	0.11921	0.3234556	0.53292
Right elbow	2 (1.79%)	2 (2.74%)	0 (0%)	1.087920	1	0.29693	0.42278	0.0038946	0.68345
Left elbow	2 (1.79%)	2 (2.74%)	0 (0%)	1.087920	1	0.29693	0.42278	0.0038946	0.68345
Right wrist	1 (0.89%)	0 (0%)	1 (2.56%)	0.052528	1	0.46860	0.65766	0.0775966	0.68345
Left wrist	1 (0.89%)	1 (1.37%)	0 (0%)	0.539059	1	0.46282	0.65179	0.0000669	0.68345
Right ankle	1 (0.89%)	0 (0%)	1 (2.56%)	0.525280	1	0.46860	0.65766	0.0775966	0.68345
Left ankle	0 (0%)	0 (0%)	0 (0%)	-	-	-	-	-	-
Right forearm	0 (0%)	0 (0%)	0 (0%)	-	-	-	-	-	-
Left forearm	1 (0.89%)	1 (1.37%)	0 (0%)	0.539059	1	0.46282	0.65179	0.0000669	0.68345
Numbness of the fingers in the left limb	1 (0.89%)	0 (0%)	1 (2.56%)	1.888658	1	0.16935	0.34821	0.0775966	0.68345
Numbness of the fingers in the right limb	0 (0%)	0 (0%)	0 (0%)	-	-	-	-	-	-
Pain in both lower limbs	1 (0.89%)	0 (0%)	1 (2.56%)	1.888658	1	0.46860	0.34821	0.0775966	0.68345
Vertex	5 (4.46%)	2 (2.74%)	3 (7.69%)	1.461891	1	0.22663	0.22808	0.2378500	0.68345
Right buttock	4 (3.57%)	4 (5.48%)	0 (0%)	2.216134	1	0.13657	0.17525	0.1047901	0.61922
Left buttock	1 (0.89%)	1 (1.37%)	0 (0%)	0.539059	1	0.46282	0.65179	0.0000669	0.68345
Right upper limb	2 (1.79%)	2 (2.74%)	0 (0%)	1.087920	1	0.29693	0.42278	0.0038946	0.68345
Left upper limb	1 (0.89%)	0 (0%)	1 (2.56%)	1.888658	1	0.16935	0.34821	0.0775966	0.68345
Right calf	3 (2.68%)	2 (2.74%)	1 (2.56%)	0.003007	1	0.95626	0.72257	0.0014183	0.72257
Left calf	1 (0.89%)	1 (1.37%)	0 (0%)	0.539059	1	0.46282	0.65179	0.0000669	0.68345
Glabella	2 (1.79%)	1 (1.37%)	1 (2.56%)	0.206712	1	0.64936	0.57722	0.0353574	0.68345
Head	11 (9.82%)	7 (9.59%)	4 (10.26%)	0.012782	1	0.90998	0.57587	0.0381846	0.68345
Right ear	1 (0.89%)	0 (0%)	1 (2.56%)	1.888658	1	0.16935	0.34821	0.0775966	0.68345
Left ear	1 (0.89%)	0 (0%)	1 (2.56%)	1.888658	1	0.16935	0.34821	0.0775966	0.68345
Throat	2 (1.79%)	2 (2.74%)	0 (0%)	1.087920	1	0.29693	0.42278	0.0038946	0.68345
Right maxillary sinus	2 (1.79%)	1 (1.37%)	1 (2.56%)	0.206712	1	0.64936	0.57722	0.0353574	0.68345
Left maxillary sinus	2 (1.79%)	1 (1.37%)	1 (2.56%)	0.206712	1	0.64936	0.57722	0.0353574	0.68345

*p < 0.05 statistical significance, adj **p < 0.0009434 statistical significance adjusted to Bonferroni correction, (1-β) –the power of a statistical test.

A multiple linear regression models for PSS-10 and NDI estimation were developed by selecting those variables which contributed significantly to PSS-10 and NDI estimation.

### Ethical Approval

The study was conducted after obtaining the consent of the Bioethical Commission of the Medical University no. R-I-002/513/2017. Participation in the research was voluntary and anonymous. Respondents obtained exhaustive information about the purpose of the study. Students had the right to terminate their participation, at any time with no repercussions.

## Results

In the questionnaire about possible symptoms and disorders of the cervical spine in the 6 preceeding months, 25 students (22.32%) declared headaches 2–3 times a week, and 51 (45.53%) 2–3 times a month (Table 1). With respect to gender statistically significant higher frequency of headaches (2–3 times a week) was noted in women (χ2 = 5.023339, *p* = 0.02510). The test power to detect the specified effect was at a mid-range level (Fisher's Exact Unilateral Test: *p* = 0.01943, 1-β = 0.6915863). With respect to Bonferroni correction and Benjamini-Hochberg procedure, the observed differences were not statistically significant (Table 1). Stiffness of the cervical spine was recorded in 31 people (27.67%), while limitation in mobility was found in 13 (11.60%) subjects. Thirty-four students (30.35%) declared dizziness that occurred more frequently in women (χ2 = 6.344721, *p* = 0.01177). The test power to detect the specified effect was at a medium level (Fisher's Exact Unilateral Test: *p* = 0.00908, 1-β = 0.7852335) (Table 1). According to Bonferroni correction and Benjamini-Hochberg procedure, the observed differences were not statistically significant (Table 1). Balance disorders concerned 14 (12.5%) subjects (Table 1). Increased muscle tone was reported by 20 participants (17.85%). Chronic muscle fatigue was observed in 11 (9.82%) cases. Numbness of the upper limbs and nausea were noted in 18 (16.07%) students. The total of 28 respondents (25%) declared the occurrence of allergy, 48 (42.85%) difficulties with concentration, 63 difficulties focusing attention (56.25%), and 28 (25%) problems with memory (Tables 1, 2). The presence of migraine was reported by 19 (16.96%) students. Pain in the temporomandibular joint was found in 33 (29.46%) people, while grinding of the teeth was noted in 30 (26.78%) subjects (Table 2). Pain in the area of temporal and masseter muscles was reported by 18 (16.07%) and 22 (19.64%) students, respectively (Table 2). Up to 14 (12.5%) respondents indicated symptoms in the occipital area (Table 2). In terms of gender statistically significant higher prevalence of pain in the back of the head was noted in women (χ2 = 5.973413, *p* = 0.01452). The test power to detect the specified effect was at a mid-range level (Fisher's Exact Unilateral Test: *p* = 0.01774, 1-β = 0.6797027) (Table 2). The observed differences were not statistically significant with respect to Bonferroni correction and Benjamini-Hochberg procedure (Table 2). As many as 86 of the respondents (76.78%) declared the occurrence of stress, 11 (9.82%) suffered from depression, and 31 (27.67%) noted mood disorders (Table 2). A higher prevalence of stress was observed in women (Pearson's Chi-Square test: χ2 = 5.399807, *p* = 0.02014; Fisher's Exact Unilateral Test: *p* = 0.01774, 1-β = 0.6797027) (Table 2). There were no statistically significant differences with respect to Bonferroni correction and Benjamini-Hochberg procedure (Table 2).

The bodychart revealed the presence of pain within the cervical spine in 53 (47.32%) respondents (Table 3). 35 (31.25%) people declared discomfort in the suboccipital area. Twenty-one students (18.75%) indicated disorders in the thoracic segment, and 52 (46.42%) in the lumbar region (Table 3). Complaints within the right and left temporal muscle were reported by 34 (30.35%) people with significantly higher prevalence observed in women (χ2 = 4.357683, *p* = 0.03684; χ2 = 6.344721, *p* = 0.01177, respectively). The test power to detect the specified effect was at a mid-range level (Fisher's Exact Unilateral Test: *p* = 0.02851, 1-β = 0.6157159; *p* = 0.00908, 1-β = 0.7852335, respectively) (Table 3). With respect to Bonferroni correction and Benjamini-Hochberg procedure, the observed differences were not statistically significant (Table 3). Numerous patients suffered from pain in the right and left shoulder girdles (23.21%, 16.96%, respectively). Head pain was found in 11 (9.82%) students (Table 4). In terms of other pain locations, the number of cases ranged from of 0–10 (Table 4).

In 37 (33.03%) students, including 22 (30.13%) women, and 15 (38.46%) men no chronic pain was found (Table 5). In 64 (57.14%) people a low intensity of chronic pain without functional disorders was noted. A high number of complaints without any accompanying dysfunctions was observed in 10 subjects (8.92%). Moderate functional limitation was noted in 1 (0.89%) person. Within the entire study group there were no disturbances of IV° (Table 5). With respect to gender, there were no statistically significant differences between all degrees of chronic pain (*p* > 0.05) (Table 5).

**Table 5:** Graded Chronic Pain Scale version 2.0 (GCPS v. 2.0) noted in the entire study group (*n* = 112), the group of women (*n* = 73) and the group of men (*n* = 39).

					**Comparison with respect to gender**			
					**Between grades 0-IV**	**Grade 0 to grades I-IV**		
**GCPS v.2**	**Classification**	**Entire** **study group**	**Group** **of women**	**Group** **of men**	**Two-Tailed Fisher's Exact Test**	**Pearson Chi-Square test** **(χ2 test)**		
		***n* = 112**	***n* = 73**	***n* = 39**	***p*-value**	**Chi^**2**^**	**df**	***p*-value**
Grade 0	No pain in prior 6 months	37 (33.04 %)	22 (30.14%)	15 (38.46%)	0.83965	0.796277	1	0.37221
Grade I	Low Intensity Low Disability	64 (57.14%)	43 (58.90%)	21 (53.85%)				
Grade II	High Intensity Low Disability	10 (8.93%)	7 (9.59%)	3 (7.69%)				
Grade III	High Disability Moderately Limiting	1 (0.89%)	1 (1.37%)	0 (0%)				
Grade IV	High Disability Severely Limiting	0 (0%)	0 (0%)	0 (0%)				

*p < 0.05 statistical significance.

In 65 (58.03%) students, including 48 (65.75%) women and 17 (43.58%) men, a moderate level of stress was observed (Table 6) and 15 people reported high level of perceived stress (Table 6). Low stress intensity was observed in 32 (28.57%) subjects (16 women and 16 men) (Table 6). In relation to gender, there were no statistically significant differences between all degrees of stress (*p* > 0.05) (Table 6).

**Table 6:** The level of the stress with respect to Perceived Stress Scale (PSS-10) in the entire study group (*n* = 112), the group of women (*n* = 73) and the group of men (*n* = 39).

						**Comparison with respect to gender**					
						**Between grades 0-III**			**Grade I to grades II, III**		
**PSS-10**	**Classification**	**Reference value**	**Entire** **study group**	**Group** **of women**	**Group** **of men**	**Pearson Chi-Square test** **(χ2 test)**			**Pearson Chi-Square test** **(χ2 test)**		
			***n* = 112**	***n* = 73**	***n* = 39**	**Chi^**2**^**	**df**	***p*-value**	**Chi^**2**^**	**df**	***p*-value**
Grade I	Low stress	0–13	32 (28.57%)	16 (21.91%)	16 (41.02%)	5.577153	2	0.06151	4.547664	1	0.03296*
Grade II	Moderate stress	14–26	65 (58.03%)	48 (65.75%)	17 (43.58%)						
Grade III	High perceived stress	27–40	15 (13.39%)	9 (12.32%)	6 (15.38%)						

*p < 0.05 statistical significance.

In 60 (53.57%) patients, mild cervical spine disorders were found (Table 7). In 3 (2.68%) subjects, moderate dysfunction was noted. Within the entire study group, no grade IV and V of disorders were observed. 49 (43.75%) respondents revealed no dysfunctions connected with in the NDI (Table 7). With respect to gender, statistically significant differences were noted between all degrees of NDI (*p* < 0.05) (Table 6).

**Table 7:** Neck Disability Index (NDI) in the entire study group (*n* = 112), the group of women (*n* = 73) and the group of men (*n* = 39).

						**Comparison with respect to gender**			
						**Between grades I-V**			**Grade I to grades II-V**		
**NDI**	**Classification**	**Reference value**	**Entire** **group**	**Group** **of women**	**Group** **of men**	**Two-Tailed Fisher's Exact Test**	**Pearson Chi-Square test** **(χ2 test)**		
			***n* = 112**	***n* = 73**	***n* = 39**	***p*-value**	**Chi^**2**^**	**df**	***p*-value**
Grade I	No disability	0-4	49 (43.75%)	25 (34.24%)	24 (61.53%)	0.01547^*^	7.693713	1	0.00554^*^
Grade II	Mild disability	5-14	60 (53.57%)	45 (61.65%)	15 (38.47%)				
Grade III	Moderate disability	15-24	3 (2.68%)	3 (4.10%)	0 (0%)				
Grade IV	Severe disability	25-34	0 (0%)	0 (0%)	0 (0%)				
Grade V	Complete disability	≥35	0 (0%)	0 (0%)	0 (0%)				

* *p < 0.05 statistical significance*.

**Figure 2:**
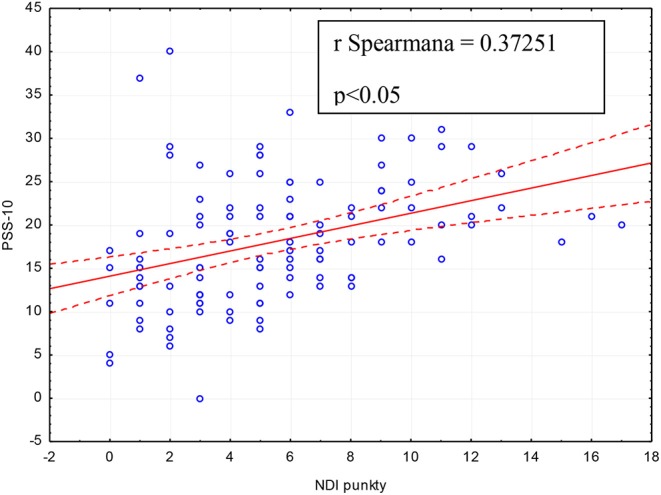
Relationship between Neck Disability Index (NDI) and Perceived Stress Scale (PSS-10) in the entire study group (*n* = 112).

The results of this study showed a direct proportional relationship between the neck disability index (NDI) and the scale of perceived stress PSS-10 (*p* < 0.05, *r* = 0.37251) (Figure 2).

The multiple linear regression showed that Neck Disability Index (NDI), pain within right masseter muscle, migraine and pain within left temporomandibular joint allow to explain about 26% differentiation of perceived stress scale (PSS-10) (*R*^2^ = 0.2635), and the prediction model is significantly better than random [*F*_(4, 107)_ = 9.5705; *p* < 0.00000], where the average error in estimating the level of perceived stress is S.E. = 6.28 (Table 8).

**Table 8:** Multiple linear regression model with PSS-10 as the dependent variable and Neck Disability Index, pain within right masseter muscle, migraine and pain within left temporomandibular joint as independent variables.

	**Regression coefficient (b)**	**SE**	**Standardized coefficient (β)**	***t*-value**	***p*-value**
Intercept	13.78894	1.06985	-	12.88869	0.00000^*^
Neck disability index	0.48146	0.17423	0.24700	2.76339	0.00674^*^
Masseter muscle right	4.79759	1.73483	0.23474	2.76546	0.00670^*^
Migraine	3.56854	1.65862	0.18727	2.15151	0.03369^*^
Temporomandibular joint left (bodychart)	4.79775	2.38821	0.17277	2.00893	0.04706^*^

R = 0.51332448.

R^2^ = 0.26350202.

Adjusted R2= 0.23596939 F(4,107)=9.5705 p < 0.00000.

Standard error of the estimate 6.2795.

SE, standard error.

* *p < 0.05 statistical significance*.

The multiple linear regression showed that gender, headache (2–3 times a month), hypersensitivity to the touch within the face, GCPS v. 2.0, PSS-10, pain within cervical spine, limitation of the mobility of the cervical spine and decrease of muscle tension allow to explain about 49% differentiation of Neck Disability Index (NDI) (*R*^2^ = 0.4943), and the prediction model is significantly better than random [*F*_(8, 103)_ = 12.5868; *p* < 0.00000], where the average error in estimating the NDI level is S.E. = 2.72 (Table 9). No interactions with age were present in any of these analyses.

**Table 9:** Multiple linear regression model with NDI as the dependent variable and gender, headache (2–3 times a month), hypersensitivity to the touch within the face, GCPS, PSS-10, pain within cervical spine cx (bodychart), limitation of the mobility of the cervical spine and muscle tension decrease as independent variables.

	**Regression coefficient (b)**	**SE**	**Standardized coefficient (β)**	***t*-value**	***p*-value**
Intercept	−0.19478	0.79027	–	−0.24648	0.80580
Gender	1.24465	0.55962	0.16161	2.22410	0.02832^*^
Headache (2–3 times a month)	1.46993	0.53752	0.19952	2.73467	0.00735^*^
Hypersensitivity to the touch within the face	7.80961	2.86231	0.20023	2.72843	0.00748^*^
Graded chronic pain scale (GCPS v. 2.0)	1.32350	0.65493	0.16967	2.02084	0.04589^*^
Perceived Stress Scale (PSS-10)	0.12425	0.03716	0.24220	3.34396	0.00115^*^
Cervical Spine Cx (bodychart)	1.40669	0.61557	0.19143	2.28518	0.02435^*^
Limitation of the mobility of the cervical spine	1.80106	0.86207	0.15724	2.08923	0.03915^*^
Muscle tension decrease	3.27915	1.62820	0.14430	2.01398	0.04662^*^

R = 0.70309427.

R^2^ = 0.49434156.

Adjusted R^2^ = 0.45506712 F_(8, 103)_ = 12.587 p < 0.00000.

Standard error of the estimate: 2.7206SE—standard error.

* *p < 0.05 statistical significance*.

## Discussion

About 70% of the world population experience neck pain at some point of their life (13). 30% of adults suffer from ailments—annually, and 5–10% lose efficiency (13). In dentists, pain occurs in the early years of their careers (13). According to Udoye, 70% of students of dentistry declare a disorders after only 3 years of studies (13, 14). Ailments mainly concern the cervical segment (74.3%) and the lumbar spine (62%) (13). Their main causes are prolonged static positions, constantly repeated movements, genetic predisposition and badly distributed lighting that provokes compensatory postures. Static positions require contraction of at least 50% of the muscles in the entire body, prolonged immobility and activity against the force of gravity (13, 15). The consequence of these is pain, as well as structural disorders, often resulting in premature termination of dentist's careers (13, 16).

According to Marklin et al. 78% dentists and 66% dental hygienists adopt a sitting position (17). For over 50% of their working time they maintain a typical bending of the trunk of at least 30°. Neck flexion at a minimum of 30° is noted by 85% after 4-h observation (3 h 24 min). For over 50% of the operative period, the cervical spine remains in a bend of 60°. The arm stays at 30° for more than 50% of the time. A static body posture dominates, with maintaining the same alignment of the shoulder and cervical spine (17). The adopted, inappropriate biomechanical system requires high energy inputs, and at the same time considerable compression in the joints conditioning the static position (17). Maintaining nonergonomic positions results in muscle pain in the area of the neck, lumbar region, and shoulder girdle. A frequent complication is also damage of the brachial rotator within the shoulder (17). It should be emphasized that the human body is not adapted to long-term static loads (18). Even after a little effort, the muscle tissue needs regeneration. On the other hand, increased static tension intensifies muscular tonus leading to blood flow disorders (18, 19). There is a local increase in the concentration of substrates and metabolites. Local changes, through afferent nerve fibers, affect the central nervous system, often leading to discomfort and fatigue. The circulatory and respiratory system is stimulated to discharge metabolites and balance the demand for oxygen and nutrients. Long-term maintenance of static positions means that blood flow may be insufficient to restore homeostasis (19). Therefore, in examined group it is possible to observe most of the surveyed symptoms and disorders, especially headache, stiffness of the cervical spine, changes of muscle tension, mood disorders, depression, stress and pain (Tables 1–4). The biopsychosocial profile is exposed.

Harvie et al. noted that chronic pain can lead to the impairment of sensory discrimination in affected areas, as well as those where there is no discomfort. The reason for these changes is probably cortical reorganization. It is typical in people with impaired sensitivity of sensation (20). Tactile skills of a dentist could be reduced. In addition to the ergonomic aspects, also individual (age, body mass index, genome, history of musculoskeletal pain), behavioral (smoking, physical activity), and psychosocial (stress level, anxiety, depression, job satisfaction) factors play an important role in the etiology of cervical pain (7).

Genebra et al. emphasized that the loss of a spouse or separation, low income and lack of knowledge, as well as the occurrence of at least two other dysfunctions (e.g., cardiorespiratory failure, gastrointestinal problems) are closely related to neck pain (7). These authors indicate that remaining in a sitting position conditioned by inappropriate posture habits, the lack of ergonomic workplace adaptation and psychosocial factors increase the activity of neck extensors and sternocleidomastoideus muscles by 35% (7). Genebra et al. drew attention to increased pressure inside the intervertebral discs, and increased compression of the tendons, joint capsules and other anatomical structures within the cervical spine (7). Such phenomena may lead to the development of inflammation within the musculoskeletal system, causing pain symptoms, neck pain included (7).

Other causes of pain in the cervical spine are injuries. The most common is “whiplash” that entails a wide spectrum of disorders. Depending on the severity of the injury, there occurs isolated cervical pain (I°), neck pain associated with musculoskeletal symptoms (II°), neck pain complicated by neurological disorders (III°), and neck pain with suspected dislocation or fracture (IV°) (21, 22). Ailments appear immediately or 48 h after the accident, and last approximately from 3 to 17 days (23). They can affect both the cervical spine and the shoulder girdle. Pain can radiate from the neck to the shoulder, occiput and even eyes. It may also spread laterally relative to the trapezius muscle (23). During the palpation of the lower segments of the cervical spine a reduced pain threshold is clinically noted. Shoulder pain occurs on one or both sides. It is more severe during arm movements (23). Dysphagia occurs secondary to the swelling of the throat and upper laryngeal nerve. Moreover, internal carotid artery stenosis may occur as well as chronic spasm of the trapezius muscle. The cranial symptoms are manifested by pain in the area of the occiput, forehead and orbit (23). Often tinnitus, visual disturbances and mood changes occur (23). The Spasm reduces the range of motion. In some cases, it is typical to maintain a cervical spine bend, because spine extension intensifies pain (23).

The consequence of spasm is myofascial pain leading to the formation of tenderness in the palpation areas of the muscle tissue. The creation of trigger points, in turn, may lead to the occurrence of transferred (projected) pain (23). Pain of the cervical spine may also result from degenerative changes in an intervertebral disc and spine joints, overloading of the cervical ligaments, torticollis, fibromyalgia, polymyalgia, rheumatoid, or ankylosing spondylitis, metabolic and infectious diseases, as well as neoplastic changes.

A chronic lack of time dictated by the scope of duties is confirmed in the Homo sedentarius model, promoting the occurrence of cardiovascular diseases, diabetes, cancer, obesity, hypertension, or depression, in addition to musculoskeletal disorders (24, 25). Hashim et al. demonstrated that the main problems for dentists with regard to systemic diseases are cardiovascular and then gastrointestinal disorders (26). The authors emphasize that only 39% of dentists regularly do sporting activities (26). On the other hand, Nalliah et al. indicated that dentists are more exposed to the occurrence of reflux, certain types of oncological diseases, backache, headache, cervical pain, osteoarthritis, including rheumatoid and psoriatic arthritis, compared to the general population (27).

This study demonstrated a quite precise of the prevalence of symptoms and disorders related to the cervical spine noted in the 6 preceeding months within the entire study group (Table 1). The most common were headaches and dizziness, stiffness, and limitation of mobility of the cervical spine, balance disturbances, increased muscle tension, nausea, numbness in the upper limbs, as well as allergies (Table 1). An interesting fact was the occurrence of concentration disorders in 42.85% of the subjects, problems with memory (25%), difficulties with focusing attention (56.25%), and myopia (41.96%) (Table 1). Perhaps this is the effect of increased muscle tension in the suboccipital area, and the resulting changes in the functioning of the dura mater and myofascial back line (28–30). Lennerstrand et al. showed that irritation of the suboccipital region may lead to visual disturbances, including the position of the eyeball (31). Similar results were obtained by Han et al. (32). Diaz-Caballero et al. underlined that in dentists, excessive or total lack of lighting is the main cause of myopia and irreversible changes in the retina (33). Our respondents reported complaints about the temporomandibular joint, pain of temporal and masseter muscles, which could indicate the presence of severe stress and emotional problems (Table 1) (34). An important role may be played by bruxism and other oral parafunctions. The psychoemotional aspect can be confirmed by mood disorders, stress, depression and gastric problems noted within the study group (Table 1).

A total of 66.97% of the respondents declared suffering from chronic pain (Table 2). The majority of students showed the disorder of I ° (57.14%), and 8.92% reported type II dysfunction. It is comforting that within the studied group there were no functional limitations to a severe degree (IV°), and only one person (0.89%) declared the moderate type (III°) concerned only one person (0.89 omen and men were comparable (Table 2). The results observed for the both groups: of women and men were comparable (Table 2).

Hasan et al. demonstrated disorders of I° in 49%, of II° in 31%, and of III° in 9% of the studied population (35). The authors did not report any type IV dysfunctions (35). Hasan et al. emphasized that the incidence of neck pain in the population of dentistry students (*n* = 400) is 21.8%. Ailments in the area of the right and left shoulder girdle are at the level of 5.1 and 3.7%, respectively. Pain in the lumbar spine was observed by 21.2% of the respondents, and 10.7% declared ailments in the thoracic segment. Pain within the elbow affected 0.6% of the students, while hand and wrist complaints were received in 3.7% of respondents. 55% of the subjects reported discomfort in the thigh or hip area, and up to 6.2% in the ankle. Pain of the lower limbs was reported by 21.2% of the students (35). In the presented study cervical ailments in relation to the body chart were observed in 47.32% of the participants (Table 2). There was also a higher incidence of left and right shoulder girdle complaint −23.21 and 16.96%, respectively. Ailments of the lumbar region were found in 46.42% of the respondents, and chest discomfort was observed in 18.75%. Elbow pain was reported by 1.78% of the subjects, and wrist complaints by 0.89%. Pain in the lower limbs was indicated by 0.89% of the students. No participant pointed to problems in the ankle area (0%) (Table 2).

The disproportion in the frequency of particular ailments in both populations may result from cultural differences, preserved or unpreserved ergonomics, as well as differences in the size of both groups. The specific profile of disorders in numerous cases may indicate the phenomenon of central sensitization of pain which may be triggered by increased emotional tension. 58.03% of the respondents declared a moderate level of stress (Table 4). Its high intensity was recorded in 13.39% of the students, while low stress intensity was observed in only 28.57% (Table 4). Beurskens et al. emphasize the biopsychosocial nature of cervical spine pain (36). The researchers describe the nature of the connections between ailments and stress, depression, frustration, anxiety, or uncertainty (36). A multifactorial etiology may be confirmed by the low relationship between the Neck Disability Index and Perceived Stress Scale (Figure 2). Alzahem et al. report that the main factors responsible for stress among dentistry students include: problems arising from accommodation, individual, environmental, academic, clinical and education-related factors (37). This authors draw attention to the key role of financial problems, restrictions on free time, lack of time for relaxation or social relations, as well as personal and family problems (37). Cooper et al. consider the profession of dentistry the most stressful of all the medical professions (38).

Regarding the Neck Disability Index (NDI), 53.57% of students had II° dysfunction (Table 6). Moderate disorders were recorded in 2.68% of the respondents (Table 6). There were no type IV or V abnormalities within the study group (Table 6). The presented disorder profile may indicate an upper crossed syndrome (UCS) or the so-called text neck (39). Upper crossed syndrome is the result of an imbalance between hypertonic and tonic muscles. The clinical picture is dominated by deepened cervical lordosis, gothic neck, protraction and shoulder elevation, as well as extension in the C1-C2 segments. Moving the body axis in the anterior direction means that maintaining the correct position of the body in the sagittal plane requires high expenditure of energy. Constant tension of the upper part of the trapezius muscle leads to the loss of function associated with movements of the skull and arms. All energy is used to maintain the correct position of the head. The sternocleidomastoideus muscle is responsible for the dislocation of the skull from side to side and nodding movements (40).

It should be emphasized that proper head position in space is related to the angle between the top of the spinous of C7 spine process, the tragus point within the ear and the horizontal plane. Under normal conditions, it is 50–55°. This angle is reduced in head protraction. Kendall et al. described the relationship between pain in the cranio-cervical area and the position of the skull and shoulders (41). Braun et al. reported that postural disorders, including cervical spine dysfunction, are factors promoting the occurrence of pain within temporomandibular joints (42).

In upper crossed syndrome there is an increased tension of the pectoralis major and minor, the descending part of the trapezius muscle, levator scapulae muscle, suboccipital muscles, sternocleidomastoid muscle, clavicular part of the deltoid muscle, and the cervical part of the erector spinae muscles. In the clinical pattern researchers observed weakness, extension, and general hypotension of deep neck flexors, the ascending and middle part of trapezius muscles, musculus serratus anterior, rhomboid muscles, as well as the spinal part of the deltoid muscle (40).

This study leads to new perspectives regarding the interplay between cervical spine dysfunction, chronic pain and stress. Multiple linear regression model with PSS-10 (dependent variable) highlighted association between stress, NDI, migraine, pain within right masseter muscle and left temporomandibular joint (Table 8). This model indicated comparable relationship of NDI and pain of the right masseter muscle with PSS-10 (comparable standardized coefficients –β and *p*-value) (Table 8). From clinical point of view, increased activity of the suboccipital muscles expressed by changes of NDI can lead to the headache, hyperactivity, and pain of the masseter muscle. Pain in the left temporomandibular joint may reflect to the contralaterality of human preference for using the dominant body part or may indicate susceptibility to the occurrence of asymmetrical craniomandibular descending dysfunction.

Multiple linear regression model with NDI (dependent variable) emphazized significant role of gender, headache (2–3 times a month), hypersensitivity within the face, GCPS, PSS-10, pain within cervical spine, limitation of the mobility of the cervical spine, and muscle tension decrease. Regression coefficients indicated independent contributions of each factor to the global evaluation of NDI (Table 9). PSS-10 showed stronger association with NDI than GCPS (higher standardized coefficients –β and *p*-value) (Table 9). NDI was also more strongly connected with hypersensitivity to the touch than with gender, headache, GCPS, pain, and limitation of the mobility of the cervical spine and muscle tension decrease.

In this model hypersensitivity could be associated with dysfunction of the trigeminal nerve and/or increased muscle tension in the suboccipital area, and thus changes in the functioning of the dura mater and myofascial back line.

Lack of statistically significant differences in relation to Bonferroni correction and Benjamini-Hochberg procedure means that there is a good chance that statistically significant differences without correction are false positive (Tables 1–4). On the other hand, cost of a false negative could mean missing an important discovery. Further research is needed.

This study emphasizes that in addition to periodic monitoring of the respondents, it would be advisable to implement preventive actions aimed at increasing health awareness among the studied group of students. Activities promoting regular physical activity as well as those aimed at improving the general physical condition are advisable, similar to the consolidation of ergonomic principles, including those specific to the profession.

## Conclusion

The relatively high prevalence of symptoms of the cervical spine dysfunction, chronic pain, stress, and neck disability among young people may indicate that numerous spinal disorders identified in dentists result not only from their profession, in which spine is significantly overloaded, but also individual predispositions including biopsychosocial profile and the changing lifestyle habits of young people.

## Data Availability Statement

Datasets are available on request. The raw data supporting the conclusions of this article will be made available by the authors, without undue reservation, to any qualified researcher.

## Ethics Statement

The studies involving human participants were reviewed and approved by Bioethical Commission of the Medical University of Bialystok, Poland no. R-I-002/513/2017. Written informed consent for participation was not required for this study in accordance with the national legislation and the institutional requirements.

## Author Contributions

JK developed and planned the study. MŻ-P contributed to the interpretation of the results and supervised the project. JK and MŻ-P participated in the writing of the manuscript. Both authors discussed the results and revised the manuscript, as well as read and approved the submitted version.

### Conflict of Interest

The authors declare that the research was conducted in the absence of any commercial or financial relationships that could be construed as a potential conflict of interest.
